# Correction: Prediction of the compressive strength of high-performance self-compacting concrete by an ultrasonic-rebound method based on a GA-BP neural network

**DOI:** 10.1371/journal.pone.0257650

**Published:** 2021-09-15

**Authors:** Guoqiang Du, Liangtao Bu, Qi Hou, Jing Zhou, Beixin Lu

There is an error in affiliation 1 for authors Guoqiang Du and Liangtao Bu. The correct affiliation 1 is: College of Civil Engineering, Hunan University, ChangSha, Hunan Province, China.
