# Supplementary figures and images for: Prediction of the compressive strength of high-performance self-compacting concrete by an ultrasonic-rebound method based on a GA-BP neural network
Source: PLoS One. 2021 May 3;16(5):e0250795. doi: 10.1371/journal.pone.0250795 (PMC8092652; doi:10.1371/journal.pone.0250795)

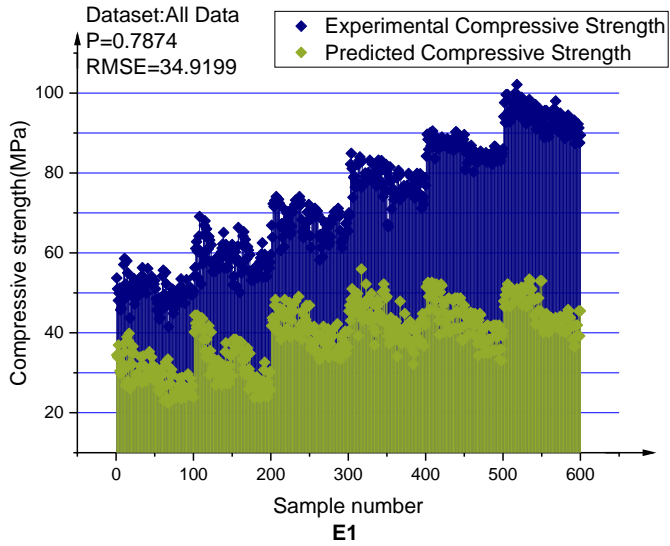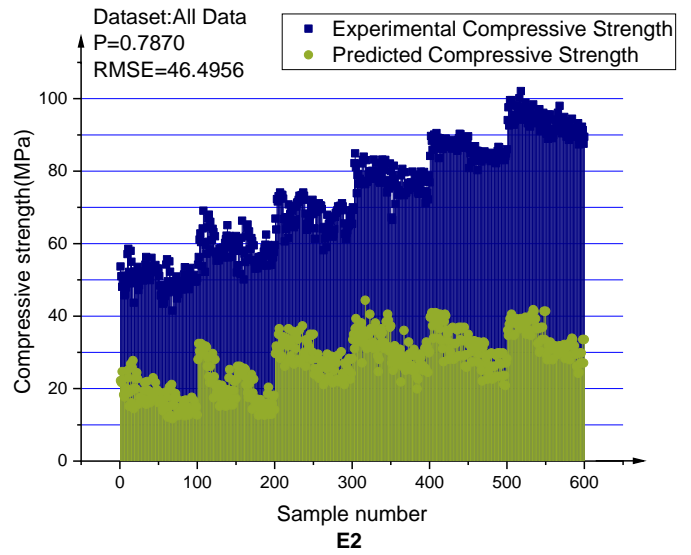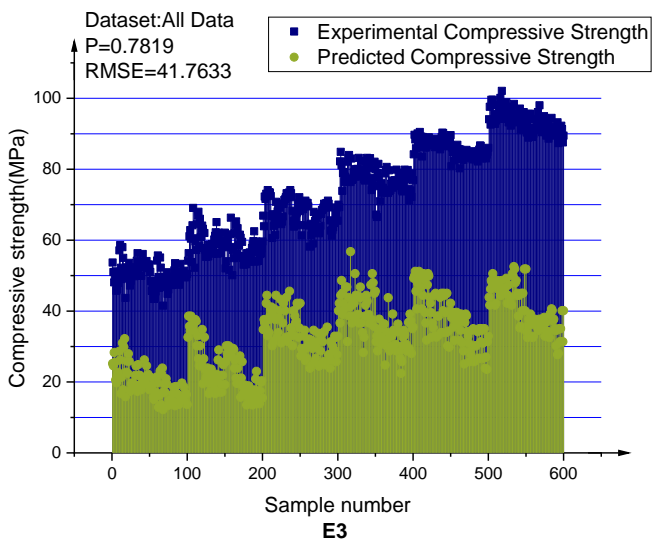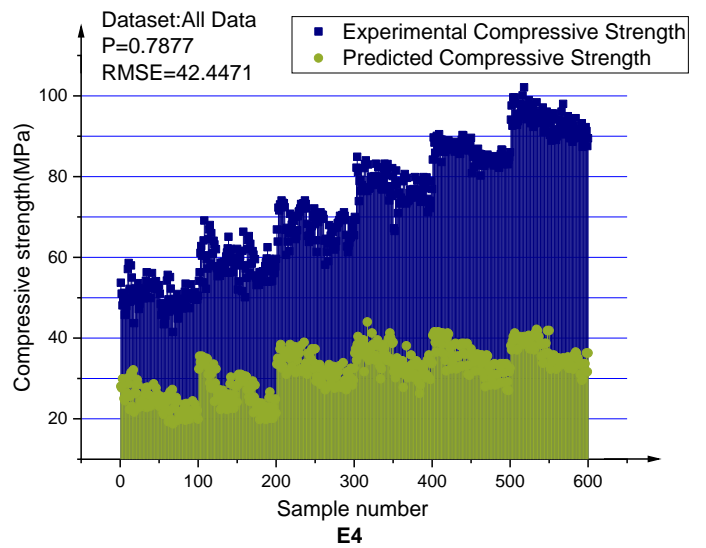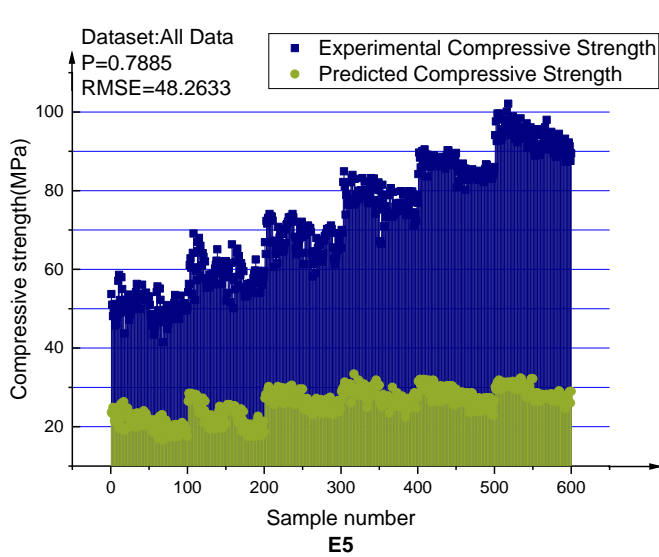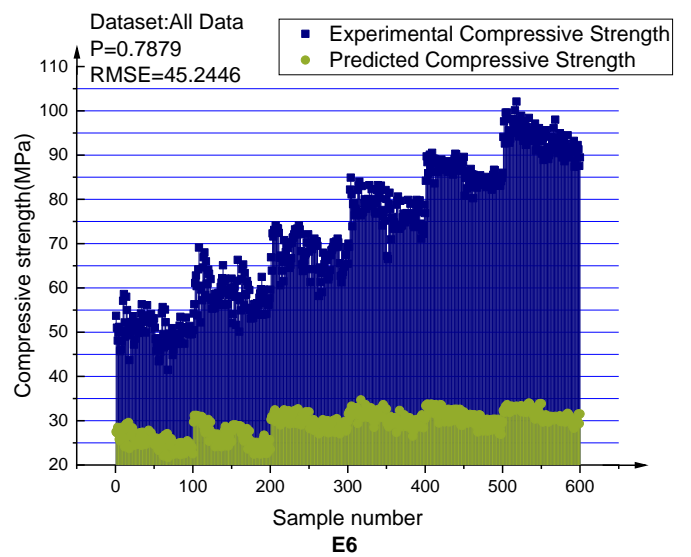

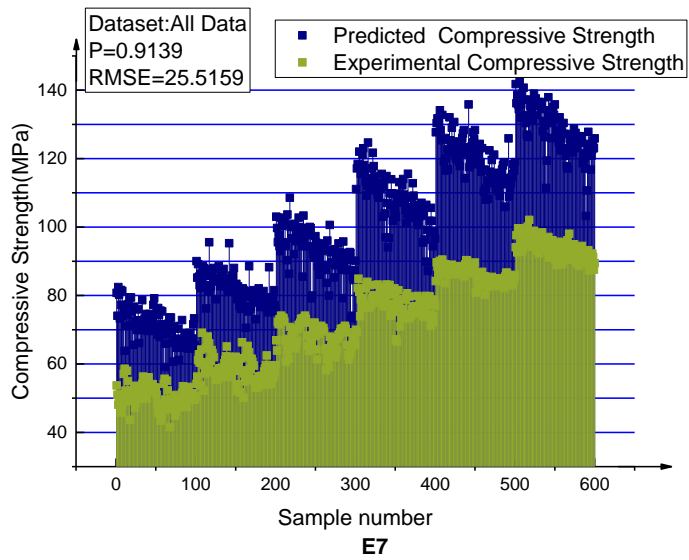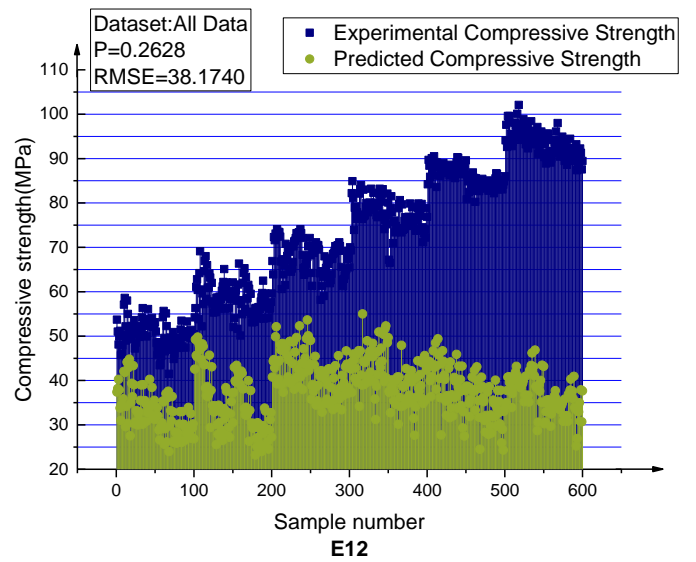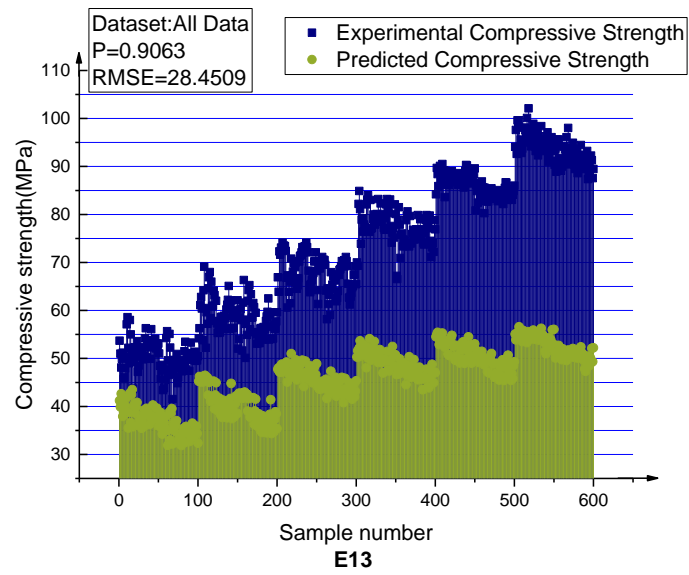

Supplement: S1 Fig — (PDF) [file pone.0250795.s001.pdf]
