# Supplementary material for: Prediction of the compressive strength of high-performance self-compacting concrete by an ultrasonic-rebound method based on a GA-BP neural network
Source: PLoS One. 2021 May 3;16(5):e0250795. doi: 10.1371/journal.pone.0250795 (PMC8092652; doi:10.1371/journal.pone.0250795)
Supplement: Attachment — Submitted filename: PONE-D-21-02136_Comments.pdf [file pone.0250795.s002.pdf]

This paper presents a genetic algorithm (GA)-optimized backpropagation neural network (BPNN) model to predict the compressive strength of self-compacting concrete using UPV and rebound value as input parameters. The content is interesting but this reviewer believes there are several critical concerns need to be carried out. Therefore, this reviewer recommends publication of this paper provided that the following major revisions are successfully carried out.

1. The originality of this paper is not clear. The authors must clearly explain what is original in this paper.
2. Please specify the chemical compositions of binders in the mix designs.
3. Section "Model development ANN" contains basically textbook contents on ANN. There is no need to give all the equations from (1) to (7). Therefore, the authors should substantially shorten this section. Please do the same for Section "Performance of the model" and equations (9) to (13).
4. There has to be more substantial discussion in Section "Results and discussion", not just providing the results.
5. Please use boxplots to show the range of your database in each set of mix designs. You can define x-axis to be the set of your six mix designs (A, B, C, D, E, F) and y-axis to be boxplots for the observed values (for instance fc) in each set of mix designs. Therefore, you have six boxplots in each plot and three plots for fc, Vp and R. Please also compare the standard deviation of each set of 100 test cubes for fc, Vp and R and discuss the reliability of your dataset.
6. R is used for both rebound value and the difference between the predicted and expected values in equations (9) to (13). Please change R for one of them.
7. In lines 279-280, *"The appropriate number of hidden-layer neurons is generally between two and 20, and usually there are one or two hidden layers."*. Please give reference.
8. In lines 339-341, *"The calculation results are shown in Fig 14. When the number of hidden-layer neurons was 14, the network had the smallest error and the model with the best performance was Model 2-19-1."*
   14 or 19?
9. Please run BPNN and GA- BPNN separately for 10 times and show the results (R2, MSE, RMSE, MAE and MAPE) in a table or plot. Then discuss the results.
10. Please discuss the limitations of your method in Conclusion.
11. Please use English language in supplementary file.
